# The Effects of Components of Fine Particulate Air Pollution on Mortality in California: Results from CALFINE

**DOI:** 10.1289/ehp.9281

**Published:** 2006-08-29

**Authors:** Bart Ostro, Wen-Ying Feng, Rachel Broadwin, Shelley Green, Michael Lipsett

**Affiliations:** 1 California Office of Environmental Health Hazard Assessment, Oakland, California, USA; 2 Graduate Group in Biostatistics, University of California, Davis, California, USA; 3 Department of Epidemiology and Biostatistics, University of California, San Francisco, California, USA

**Keywords:** EC, fine particles, mortality, nitrates, OC, particulate matter, PM_2.5_, species

## Abstract

**Objective:**

Several epidemiologic studies provide evidence of an association between daily mortality and particulate matter < 2.5 μm in diameter (PM_2.5_). Little is known, however, about the relative effects of PM_2.5_ constituents. We examined associations between 19 PM_2.5_ components and daily mortality in six California counties.

**Design:**

We obtained daily data from 2000 to 2003 on mortality and PM_2.5_ mass and components, including elemental and organic carbon (EC and OC), nitrates, sulfates, and various metals. We examined associations of PM_2.5_ and its constituents with daily counts of several mortality categories: all-cause, cardiovascular, respiratory, and mortality age > 65 years. Poisson regressions incorporating natural splines were used to control for time-varying covariates. Effect estimates were determined for each component in each county and then combined using a random-effects model.

**Results:**

PM_2.5_ mass and several constituents were associated with multiple mortality categories, especially cardiovascular deaths. For example, for a 3-day lag, the latter increased by 1.6, 2.1, 1.6, and 1.5% for PM_2.5_, EC, OC, and nitrates based on interquartile ranges of 14.6, 0.8, 4.6, and 5.5 μg/m^3^, respectively. Stronger associations were observed between mortality and additional pollutants, including sulfates and several metals, during the cool season.

**Conclusion:**

This multicounty analysis adds to the growing body of evidence linking PM_2.5_ with mortality and indicates that excess risks may vary among specific PM_2.5_ components. Therefore, the use of regression coefficients based on PM_2.5_ mass may underestimate associations with some PM_2.5_ components. Also, our findings support the hypothesis that combustion-associated pollutants are particularly important in California.

Particulate matter (PM) air pollution is ubiquitous in the urban environment, representing a heterogeneous mix of solid and liquid particles generated by many different sources. Several recent multicity time-series studies have demonstrated associations between daily mortality and fine PM [i.e., particles < 2.5 μm in aerodynamic diameter (PM_2.5_)] (e.g., Laden et al. 2000; Ostro et al. 2006). There is little information, however, about the relative effects of PM_2.5_ constituents. The National Research Council (NRC) recently highlighted the importance of investigating characteristics and constituents of particles that contribute to their toxicity (NRC 2004). Differential toxicity can have important implications for both the establishment of ambient air quality standards and for more targeted PM control strategies. Specifically, focusing regulations on the most toxic PM_2.5_ constituents could protect public health at a lower total cost.

Previous time-series analyses indicate that, of the sources of PM, motor vehicle exhaust usually has the strongest associations with all-cause or cardiovascular mortality (Janssen et al. 2002; Laden et al. 2000; Mar et al. 2000). Epidemiologic examinations of specific constituents of PM_2.5_ also indicate that elemental and organic carbon (EC and OC) and several transition metals are associated with mortality (Burnett et al. 2000; Mar et al. 2000). In California, the ambient particle chemistry, size distributions, and temporal patterns of exposure are different from those in other parts of the United States and Canada (Blanchard 2003). In previous work, we demonstrated associations of daily PM_2.5_ mass concentrations with total mortality and with several mortality subcategories in nine heavily populated California counties (Ostro et al. 2006). In 2000, the U.S. Environmental Protection Agency (EPA) and the California Air Resources Board (CARB) embarked on a program to systematically collect data on constituents of PM_2.5_ throughout much of California, providing an opportunity to examine daily measurements of these data in relation to mortality.

In this article, we report the results of our analysis of PM_2.5_ components and mortality in six counties. For comparison, we also examined associations with PM_2.5_ in a larger data set that includes nine California counties. The use of multiple cities in our analysis enhances statistical power, reduces the likelihood of spurious results from a single city, and incorporates a broader range of relevant geographic and population characteristics such as climate, background health status, demographics, and economic status.

## Data and Methods

### Mortality data

We obtained data on daily mortality for all California residents from the California Department of Health Services, Center for Health Statistics (CDHS), for the period for which data on PM_2.5_ components were collected: 1 January 2000 through 31 December 2003 (CDHS 1999–2003). We also collected mortality data from 1999 to support additional analyses of PM_2.5_ (CDHS 1999). A death was included only when it occurred in the decedent’s county of residence. Daily counts of total deaths (minus accidents and homicides) were aggregated for all ages. In addition, we determined daily total mortality counts for those > 65 years of age and for deaths from respiratory disease [*International Classification of Diseases, 10th Revision* (ICD10; World Health Organization 1993) codes J00–J98] and cardiovascular disease (codes I00–I99).

### Pollutant and meteorologic data

We obtained PM_2.5_ speciation data for the 4-year period 2000 through 2003 from the CARB (CARB 2004). The speciation monitors were part of the State and Local Air Monitoring Stations network, and were filter-based Met One Speciation Air Sampling Systems (Met One Instruments Inc., Grants Pass, OR). We included only counties with ≥ 180 days of observations with PM_2.5_ species data to ensure sufficient statistical power. Thus, our study of PM_2.5_ components was limited to deaths occurring in six California counties, which included approximately 8.7 million people, or 25% of the state’s population. Each of the six counties had two monitors measuring PM_2.5_ components and mass. In three counties (Fresno, Kern, and Riverside), the two monitors were located within four meters of each other in the cities of Fresno, Bakersfield, and Rubidoux, respectively. In the other counties (Sacramento, San Diego, and Santa Clara) the monitors were not co-located. Fresno, Kern, Riverside, and Sacramento Counties reported data every third day, whereas San Diego and Santa Clara Counties reported data every sixth day. For the speciation analyses, the number of observation days available ranged from 243 (San Diego County) to 395 (Sacramento County). The following constituents of PM_2.5_ were measured as 24-hr averages: EC, OC, nitrates (NO_3_), sulfates (SO_4_), aluminum, bromine, calcium, chlorine, copper, iron, potassium, manganese, nickel, lead, sulfur, silicon, titanium, vanadium, and zinc. These PM_2.5_ components represent multiple sources of PM_2.5_, including gasoline combustion, diesel exhaust, wood smoke, crustal material, and secondary pollutants, among others.

We also analyzed PM_2.5_ mass using a larger data set from 1999 through 2003 using all available monitors (including those that did not collect species data) for nine California counties—the same six counties as above plus Contra Costa, Los Angeles, and Orange Counties. The nonspeciated network data were obtained from the CARB (2004). PM_2.5_ monitors were filter-based samplers (model RAAS2.5–300; Thermo Andersen, Smyrna, GA). From the nonspeciated network, six counties had only one monitor each collecting daily PM_2.5_ data, whereas Los Angeles, San Diego, and Santa Clara Counties had three, three, and two monitors, respectively.

To allow adjustment for the effect of weather on mortality, we collected daily average temperature and humidity data at meteorologic stations in each of the counties. Hourly temperature data were obtained from the National Oceanic and Atmospheric Administration (NOAA) National Climatic Data Center (NCDC 2004). All daily mortality, pollutant, and meteorologic data were converted into a SAS database (SAS Institute Inc., Cary, NC) and merged by date.

### Methods

Counts of daily mortality are non-negative discrete integers representing rare events; such data typically follow a Poisson distribution. Therefore, we used Poisson regression, conditional on the explanatory variables. In the basic analytic approach, we used similar model specifications for each city, including smoothers for time trend and weather using natural splines. The natural spline model is a parametric approach that fits cubic functions joined at knots, which are typically placed evenly throughout the distribution of the variable of concern, such as time. The number of knots used determines the overall smoothness of the fit. Previous analysis has indicated that different spline models generate relatively similar results, although increasing the number of knots generally tends to decrease the estimated effect of pollution [Health Effects Institute (HEI) 2003; Ostro et al. 2006].

The basic regression model included the following time-varying covariates: day of week, smoothing splines of one-day lags of average temperature and humidity [each with 3 degrees of freedom (df)], and a smoothing spline of time with 4 df per year of data. We chose 4 df *a priori* because this number has been found to control well for seasonal and secular patterns (HEI 2003; Ostro et al. 2006). However, we conducted additional sensitivity analyses to evaluate the impact of alternative df for the smooth of time. In our primary analysis for each pollutant, we examined single-day lags of 0–3 days. Because the species data were only available every third or sixth day, multiday exposure averages could not be constructed. To facilitate comparisons of PM_2.5_ with its components, PM_2.5c_ was created. PM_2.5c_ was limited to values of PM_2.5_ mass measured at monitors that also measured PM_2.5_ components on the same days. Therefore, PM_2.5c_ measurements included data from six counties from 2000 through 2003. To maximize the PM_2.5_ measurements and our statistical power, we also developed an extended metric for PM_2.5_ (PM_2.5ext_) that used both PM_2.5c_ and any other available measurements of PM_2.5_ from 1999 through 2003 for nine (rather than the original six) California counties.

Regression models were run for each county, and the results were combined in a meta-analysis using a random-effects model (Der Simonian and Laird 1986), although results were fairly similar using a fixed-effects model.

To obtain a daily county pollutant concentration while accounting for missing data, we used the same process as that reported by Wong et al. (2001). For each species, the daily average was developed using the following method: *a*) calculating the mean value for each monitor across the study period; *b*) subtracting each monitor’s mean concentration from the nonmissing daily values for that monitor (i.e., centered data); *c*) calculating the daily mean of the available centered data across all monitors in a given county; and *d*) by day, adding back the grand mean (the mean of all unadjusted daily values of all of the monitors). On days when no data for a given pollutant were available from any monitor in the county, that day was recorded as missing; no data were imputed. Results generated using this data set involved a tradeoff between the increased sample size and statistical power and the potential effects on measurement error introduced through the use of multiple monitors in different parts of a given county.

Several sensitivity analyses were conducted. First, we examined the potential measurement error created by combining data for each county from multiple monitors with differing numbers of missing values. We created a data set limited to the single monitor within each county with the most (and at least 180) observations for PM_2.5_ mass and its components. As a second series of sensitivity analyses, we examined the effects of alternative smoothers of time, using either 3 or 6 df for time trend, as opposed to 4 df in the basic model. Third, we examined the effect of alternative specifications of temperature and humidity, using unlagged values for these covariates, as opposed to the 1-day lag used in the basic model. Finally, we stratified the data set by warm (April–September) and cool (October–March) periods to examine potential seasonal influences.

All final results were calculated using R (version 2.1.1; R Development Core Team 2004) for the single-county analyses and Stata (StataCorp 2003) for the meta-analyses. To compare relative impacts based on observed concentrations, the results are presented as the excess risk [i.e., (RR-1) × 100] in daily mortality for the interquartile range (IQR) of the pollutants. The full set of results, including the percent change in mortality per microgram per cubic meter for each component, is available online in the Supplemental Material (http://www.ehponline.org/docs/2006/9281/suppl.pdf).

## Results

Table 1 provides descriptive statistics for mortality categories, air quality, and meteorologic data from six counties with species data, as well as the other three counties included in the analysis of PM_2.5_ mass concentrations only. Mean daily mortality varied from 147 in Los Angeles County to 11 in Kern County. Mean daily PM_2.5_ concentrations over the study period averaged around 19 μg/m^3^, and ranged from 13 μg/m^3^ in Sacramento and Contra Costa Counties to 27 μg/m^3^ in Riverside County, exceeding the U.S. EPA annual average PM_2.5_ standard of 15 μg/m3 in six counties, and the California annual average standard of 12 μg/m^3^ in all nine counties. Table 2 summarizes the data on PM_2.5_ and its components for the full study period and for the cooler seasons (October–March). Over the four years, there were a total of approximately 1,870 observations across the six counties for most of the species. The largest contributors to PM_2.5_ were EC (5%), OC (37%), NO_3_ (28%), and SO_4_ (10%). Table 3 provides the correlations among the species and PM_2.5_. Moderate to high correlations (*r* = 0.4–0.6) were found between PM_2.5_ and EC, OC, NO_3_, Br, K, and Zn. More modest correlations (*r* = 0.2–0.4) were observed between PM_2.5_ and SO_4_, Ca, Cu, Fe, Pb, S, Ti, and V.

Table 4 provides a summary of the basic meta-analytic results for alternative single-day lags of pollutant concentrations. The results suggest many associations between the pollutants and the mortality end points. Among the pollutants from the speciation network, the strongest associations were observed for PM_2.5_ mass, EC, NO_3_, Cl, Cu, Fe, K, Ti, V, and Zn. Adding observations to PM_2.5_ mass by using data from the nonspeciation counties (so that all nine counties were included) enhanced the statistical power and resulted in observable associations with all four of the mortality categories. When the results by mortality end points were examined, several patterns emerged. All-cause mortality was associated most strongly with Cu and PM_2.5ext_, with weaker associations also observed with NO_3_ and Cl. Cardiovascular mortality was associated most strongly with EC, K, Zn and PM_2.5_ with more modest associations observed with OC, NO_3_, Fe, and Ti. Respiratory mortality was associated with Cu and Ti, with weaker associations with V, Zn, and PM_2.5ext_. Finally, for mortality among those > 65 years of age, significant associations were observed with PM_2.5_, NO_3_, Cl, K, and Zn.

**Figure 1:**
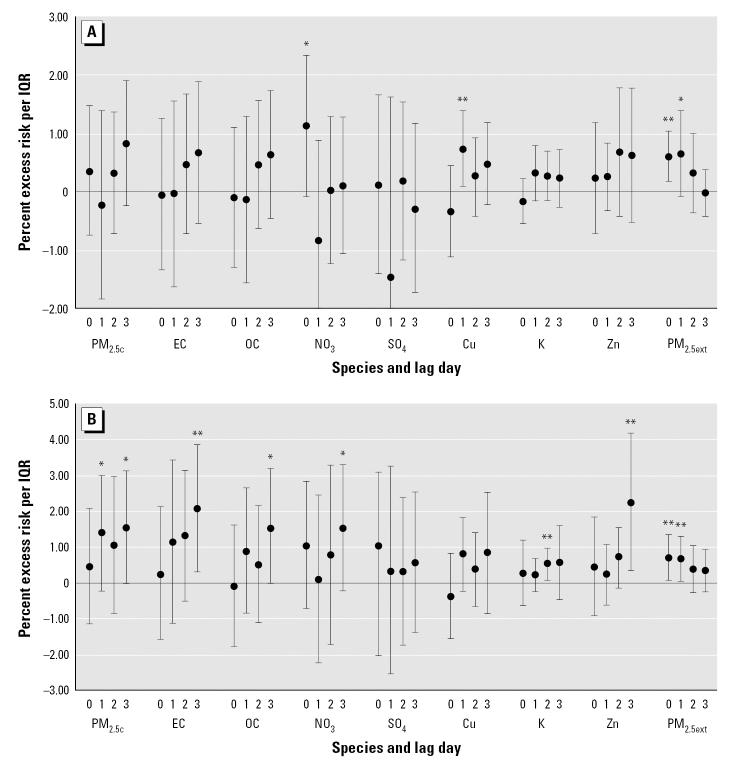
Excess risk [mean (95% CI)] of mortality per IQR of concentrations. (*A*) All-cause mortality. (*B*) Cardiovascular mortality. *p < 0.10. **p < 0.05.

Figure 1 summarizes the quantitative meta-analytic results for all-cause and cardiovascular mortality using single-day lags of selected pollutants (the full set of results is available in the Supplemental Material: http://www.ehponline.org/docs/2006/9281/suppl.pdf). Unlike many time-series studies with continuous daily data, not all lags refer to the same outcome days. Specifically, for PM data collected every third day, lags 0 and 3 will generally refer to the same days (and numbers of deaths per day) except at the ends of the time series. However, for those same PM data, lags 1 and 2 refer to different days with different numbers of deaths. Although this phenomenon holds true for other studies using nondaily PM data, the number of observations used in this analysis is small relative to those in most published studies of PM and mortality. Therefore, the results are somewhat sensitive to the specified lag; however, the findings suggest many associations between the pollutants and mortality end points. For example, for a 3-day lag, cardiovascular mortality increased by 1.6% [95% confidence interval (CI), 0–3.1] for PM_2.5_, 2.1% (95% CI, 0.3–3.9) for EC, 1.6% (95% CI, −0.1 to 3.2) for OC, 1.5% (95% CI, −0.2 to 3.3) for nitrates and 2.2% (95% CI, 0.3–4.2) for Zn for IQRs of 14.6, 0.8, 4.6, 5.5, and 0.01 μg/m^3^, respectively. Most CIs are large due to the relatively low numbers of observations. In comparing the beta coefficients, the percent change in cardiovascular mortality per microgram per cubic meter was much greater for many of the components relative to PM_2.5_ mass (see Supplemental Material: http://www.ehponline.org/docs/2006/9281/suppl.pdf). For example, the risk per unit of EC, OC, NO_3_, K, and Zn were several times higher than that of PM_2.5_ mass.

**Figure 2:**
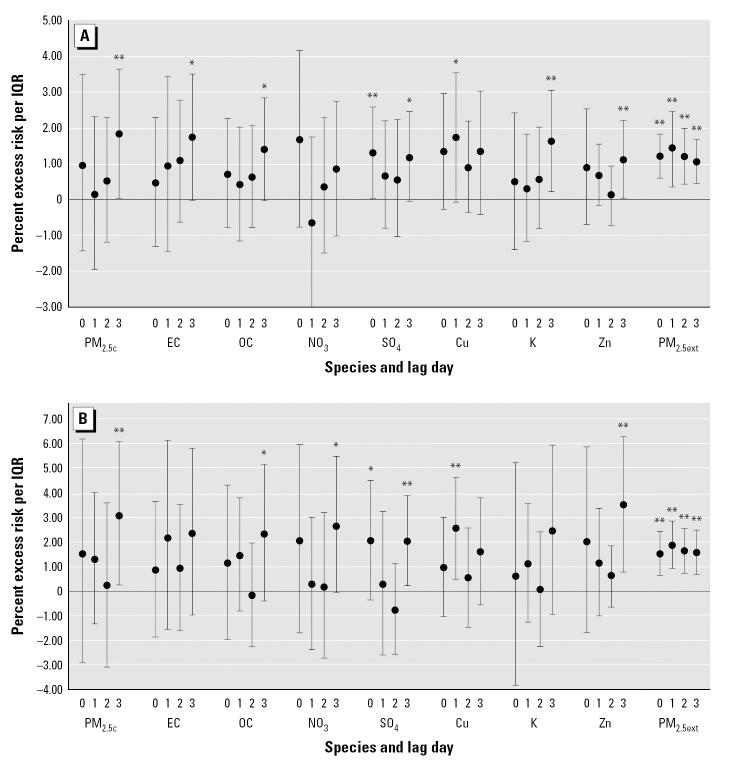
Excess risk [mean (95% CI)] of mortality per IQR of concentrations for the cooler months (October–March). (*A*) All-cause mortality. (*B*) Cardiovascular mortality. *p < 0.10; **p < 0.05.

Table 5 and Figure 2 summarize the cool season–specific results. During the cooler months, there are more associations between the pollutants and mortality than when the entire year is included in the analysis. Except for Al, Br, and Ni, almost all of the pollutants were associated with all-cause and cardiovascular mortality, and with daily deaths among those > 65 years of age. In contrast, during the summer months there were few associations, except for K with cardiovascular and respiratory deaths, and Al, Cl, Cu, Pb, Ti, and Zn with respiratory mortality (data not shown). Additional sensitivity analyses indicated that the species results were insensitive to treatment of missing values, alternative df used for the smoothers of time and weather, and different lags for the weather terms in the model specifications (data not shown).

## Discussion

In this time-series analysis of PM in California, ambient concentrations of several constituents of fine particles were associated with daily mortality. Specifically, the data suggest consistent associations with EC, OC, NO_3_, Cu, K, Ti, and Zn, as well as with PM_2.5_ mass. Stronger associations were observed with mortality for cardiovascular disease and among those > 65 years of age. For cardiovascular mortality, risks associated with the IQRs of EC and Zn were particularly elevated. Comparison of the pollution regression coefficients indicated that, in general, EC and many of the other species that contribute significantly to PM_2.5_ mass, including OC, NO_3_, and Zn, all demonstrated higher excess risks than PM_2.5_ mass. Although this observation may be partly the result of stochastic variability, the associations with mortality were all the more striking given the relatively small number of days with species data in each county (range 243–395), because most time-series studies have > 1,000 days of data (HEI 2003). Increasing the sample size increased the strength of the PM_2.5_ associations with mortality. With few exceptions, these results were relatively insensitive to alternative treatment of missing values, different smoothers of time, and different lag specifications for meteorologic covariates. Results were somewhat sensitive, however, to the lag day examined. More of the associations were with a 1-day lag, which is fairly consistent with many previous time-series studies of PM < 10 μm in aerodynamic diameter (PM_10_) and PM_2.5_ (HEI 2003). Although there is increasing evidence linking PM exposures with cardiovascular pathophysiology (Brook et al. 2004), there is little to justify *a priori* an appropriate lag structure for the vast majority of PM_2.5_ constituents. In this analysis, it is unclear whether the associations of mortality with different lags were caused by *a*) different mechanisms; *b*) different mortality reference days for lags 1 and 2 versus lags 0 and 3 because the exposure data were not collected on a daily basis (see “Methods”); or *c*) stochastic variability due to the relatively low number of observations.

We found stronger and more frequent associations between mortality and PM_2.5_ components during the cooler months, when most (but not all) components have higher concentrations. For example, the warm and cool season averages for PM_2.5_ were 14 and 24.6 μg/m^3^, respectively. For EC, OC, and NO_3_, the cool season averages were roughly twice those of the warm season. These differences represent seasonal variation in sources (e.g., residential wood combustion), particle chemistry and meteorology. For example, Lipsett et al. (1997) reported that during the winter in Santa Clara County, residential wood combustion accounts for as much as 45% of PM _1 0_ . Moreover, in the winter months the inversion layers and vertical mixing depths throughout much of the state tend to be much shallower than in warmer months. In addition, the generally mild climate in California in the cooler months may mean that more windows are open, resulting in greater indoor penetration of outdoor pollutants relative to the summer months when air conditioner use is more common.

Becker et al. (2005) reported seasonal variation in the toxicity of PM, based on *in vitro* analysis of markers of inflammation and oxidative stress, which they hypothesized could be explained by temporal differences in particle composition. In our analysis, evidence of seasonally different effects for a specific PM_2.5_ species suggests that differences in both composition and exposure patterns may be important. Our findings differ somewhat with the time-series mortality analysis of PM_10_ in 100 U.S. cities by Peng et al. (2005). That study reported stronger effects during the summer months, based on observations from cities primarily in the Northeast. Their base case region–specific analysis showed a modest warm season effect for the Northwest (which included Northern California) but no season-specific effect for Southern California, the only region that did not show a larger effect in summer in their analysis. However, the latter results appear to be sensitive to the df in the smooth of time and the PM_10_ lag used. For example, if 3 or 5 df for time smooth or an unlagged PM_10_ was specified, the effects were larger in the nonsummer months, a result consistent with our findings. In comparison, our results were not affected by use of alternative df for the smooth of time, but were sensitive to the specified lag of pollution.

A few previous studies have examined the associations between some species of PM and daily mortality. For example, Fairley (2003) examined the impacts of NO_3_, SO_4_, and coefficient of haze (COH) in Santa Clara County. The latter is highly correlated with EC, and is likely to be a good marker of particulate pollution from motor vehicles, especially diesel exhaust, and from wood smoke. All three PM_2.5_ constituents were associated with all-cause mortality, whereas NO_3_ was also associated with cardiovascular mortality. These findings were consistent with those of Hoek (2003) in the Netherlands, where associations with mortality were reported for SO_4_, NO_3_, and black smoke. In a study in Buffalo, New York, Gwynn et al. (2000) reported associations of COH, SO_4_, and hydrogen ion (a measure of aerosol acidity) with total mortality. Ito (2003) failed to find associations of mortality with SO_4_ or hydrogen ion in Detroit, Michigan, although only limited data for these pollutants were available. In their study of the eight largest Canadian cities, Burnett et al. (2000) examined the impact of 47 separate constituents of PM_2.5_. Within the fine fraction, SO_4_, Zn, Ni, and Fe were all associated with mortality, as was COH. NO_3_, EC, and OC were not measured in the Canadian study. Mar et al. (2000) reported associations between mortality in Phoenix, Arizona, and EC, OC, and K. Finally, in analyses of emergency department visits, Metzger et al. (2004) reported associations of both EC and OC with visits for any cardiovascular disease. Several other studies also examined source-oriented combinations of pollutants. For example, Laden et al. (2000) examined PM_2.5_ data from the Harvard Six Cities study and categorized the pollutants as motor vehicle exhaust (using Pb as a marker), coal combustion (using selenium), or soil and crustal material (using Si). Generally, both the motor vehicle and coal factors were associated with mortality, with the strongest effect from the former. The crustal material indicator was not associated with mortality. In our full-year analysis, we also found no association with Si; however, associations with mortality were observed during the cooler months. Factor analysis of multiple elements conducted by Mar et al. (2000) in Phoenix suggested associations between cardiovascular mortality and factors relating to three sources: motor vehicle exhaust and resuspended road dust; vegetative burning; and regional SO_4_.

Our findings are generally consistent with these previous findings—primary and secondary products of fuel combustion (EC, OC, NO_3_, as well as SO_4_ in the winter) and other measures of mobile source-related emissions (Cu, Ti, and Zn) exhibit the strongest and most consistent associations with mortality. However, we have not undertaken a formal source apportionment analysis in this paper. Although EC and OC are generally considered markers of fossil fuel combustion, residential wood combustion may also make significant contributions to both of these as well (Lewis et al. 2003; Maykut et al. 2003). A variety of vehicular-associated sources, including fuel combustion, may contribute to particulate metal emissions, including Cu, Ti, Zn, and V (Schauer et al. 2006). For instance, brake wear and lube oil emissions may represent important sources of fine particulate Cu and Zn, though the latter may also be associated with tire dust (Lough et al. 2005; Schauer et al. 2006). Although potassium is a crustal element, it is generally considered a reasonable marker for vegetative burning, including residential wood combustion (Maykut et al. 2003; Watson et al. 2001).

The use of multiple cities increased the statistical power and reduced the likelihood that these results were due to factors associated with one geographic location. The association with mortality of any single substance, however, may be a result, at least in part, of its own toxicity or of exposures to other substances with which it is highly correlated. In future work, we will examine the impact of specific sources through use of source profiles for the six California counties based on chemical mass balance models (Thurston et al. 2005).

It is important to note the limitations of our data. First, the use of a single location for monitoring PM_2.5_ components in several of the counties is likely to lead to random measurement error and the potential for down-wardly biased effect estimates. Second, because every-day monitoring was not available, we were unable to estimate the impact of cumulative exposures, which tend to generate larger effect estimates than that of a single-day lag (Schwartz 2000). Third, given the numbers of pollutants and end points examined and the relatively low number of observations, it is possible that some of the results may have occurred by chance. Finally, there may be differential measurement error among the components both with respect to spatial variability and indoor/outdoor penetration. For example, Janssen et al. (2005) analyzed the longitudinal correlation of personal and outdoor concentrations of several PM_2.5_ species in Helsinki, Finland, and Amsterdam, the Netherlands. Correlations were high (0.7–0.9) for EC (using an absorption coefficient) and Zn; moderate (0.55–0.7) for PM_2.5_, Fe, and K; and low (0.25–0.55) for Ca, Cl, Cu, and Si. The authors suggest that these differences could be a result of both ambient spatial variability and the influence of indoor generation of pollutants.

Our findings add to the growing body of evidence linking PM_2.5_ with mortality and indicate that excess risks may vary with the specific PM_2.5_ constituent. The results also support the hypothesis that pollution from motor vehicles and other sources of combustion, including residential wood burning, may be of particular concern. Finally, the use of regression coefficients based only on PM_2.5_ mass may underestimate the effects of some of its specific components.

## Figures and Tables

**Table 1:** Mean daily deaths by mortality category and air quality and meteorologic data, by county, 2000–2003.

	Contra Costa	Fresno	Kern	Los Angeles	Orange	Riverside	Sacramento	San Diego	Santa Clara
Mortality category									
All causes	15.7	13.4	11.4	146.7	40.2	28.8	22.0	49.5	21.3
Cardiovascular disease	6.4	5.8	5.1	66.3	17.8	13.2	9.3	20.3	8.7
Respiratory disease	1.8	1.5	1.5	15.1	4.3	3.4	2.6	5.5	2.4
Age > 65 years	12.2	10.1	8.4	108.6	31.8	22.8	16.3	38.8	16.4
County characteristics									
Population (1,000s)	949	799	662	9,519	2,846	1,545	1,223	2,814	1,683
Mean PM_2.5_ (μg/m^3^)	12.8	17.5	19.5	20.8	21.5	27.1	12.6	15.3	13.9
Mean temperature (°F)	60.1	64.2	65.7	63.8	63.6	65.5	61.8	61.9	59.5
Mean relative humidity	64.4	56.5	58.2	58.8	71.8	62.6	66.1	75.8	68.3
Days in species analysis	0	355	281	0	0	279	395	243	317

**Table 2:** Descriptive statistics for PM_2.5_ and species in California counties, 2000–2003.

	All-year					Cooler months (Oct–Mar)		
Pollutants	Obs^a^	Mean (μg/m^3^)	IQR (μg/m^3^)	95^th^ percentile	% Below detection	Obs^a^	Mean (μg/m^3^)	IQR (μg/m^3^)
PM_2.5c_^b^	1,878	19.28	14.63	46.91	0.00	844	24.60	21.47
EC	1,879	0.966	0.795	2.57	0.05	842	1.319	1.135
OC	1,879	7.129	4.592	15.91	0.00	842	9.192	6.124
NO_3_	1,817	5.415	5.524	17.46	0.00	831	7.294	7.985
SO_4_	1,817	1.908	1.530	5.18	0.00	831	1.483	1.233
Al	1,870	0.044	0.051	0.14	5.45	845	0.036	0.042
Br	1,870	0.004	0.004	0.01	1.76	845	0.005	0.004
Ca	1,870	0.080	0.064	0.20	0.05	845	0.081	0.066
Cl	1,870	0.094	0.069	0.41	2.09	845	0.105	0.083
Cu	1,870	0.007	0.007	0.34	1.34	845	0.008	0.008
Fe	1,870	0.124	0.099	0.26	0.00	845	0.138	0.117
K	1,870	0.117	0.081	0.01	0.00	845	0.135	0.112
Mn	1,870	0.003	0.003	0.01	3.21	845	0.003	0.003
Ni	1,870	0.005	0.003	0.01	2.51	845	0.005	0.003
Pb	1,870	0.004	0.004	1.70	2.83	845	0.005	0.005
S	1,870	0.648	0.499	0.43	0.00	845	0.522	0.434
Si	1,870	0.168	0.151	0.04	0.05	845	0.147	0.132
Ti	1,870	0.009	0.008	47.69	0.32	845	0.008	0.008
V	1,870	0.002	0.003	46.91	5.56	845	0.002	0.002
Zn	1,870	0.012	0.011	2.57	1.82	845	0.018	0.014
PM_2.5ext_^c^	11,494	18.6	15.1	15.9	0.00	5,777	22.40	19.8

a Total number of observations (Obs) for analysis across all of the counties.

b Includes six counties with species data, 2000–2003.

c Includes all nine counties in Table 1, 1999–2003.

**Table 3:** Longitudinal correlations of PM_2.5_ and its components.

	PM_2.5c_^a^	EC	OC	NO_3_	SO_4_	Al	Br	Ca	Cl	Cu	Fe	K	Mn	Ni	Pb	S	Si	Ti	V	Zn	PM_2.5ext_^b^
PM_2.5c_^a^	1																				
EC	0.53	1																			
OC	0.62	0.61	1																		
NO_3_	0.65	0.41	0.44	1																	
SO_4_	0.32	0.05	0.12	0.35	1																
Al	0.02	0.05	0.05	−0.06	0.07	1															
Br	0.54	0.41	0.45	0.45	0.36	0.08	1														
Ca	0.23	0.27	0.17	0.11	0.19	0.29	0.31	1													
Cl	0.15	0.10	0.08	0.08	−0.07	−0.08	0.05	−0.02	1												
Cu	0.23	0.29	0.26	0.15	0.10	0.11	0.26	0.23	−0.04	1											
Fe	0.38	0.48	0.39	0.23	0.16	0.32	0.41	0.61	−0.01	0.32	1										
K	0.52	0.48	0.57	0.34	0.09	0.13	0.41	0.27	0.13	0.26	0.41	1									
Mn	0.21	0.24	0.18	0.14	0.13	0.32	0.21	0.28	0.04	0.11	0.39	0.20	1								
Ni	0.11	0.08	0.02	0.10	0.16	0.07	0.10	0.21	0.03	0.04	0.20	0.03	0.13	1							
Pb	0.27	0.28	0.27	0.22	0.06	0.03	0.23	0.10	0.12	0.11	0.19	0.23	0.13	0.04	1						
S	0.35	0.07	0.14	0.36	0.85	0.08	0.38	0.19	−0.05	0.12	0.18	0.12	0.13	0.15	0.08	1					
Si	0.16	0.19	0.15	0.05	0.20	0.44	0.28	0.62	−0.15	0.23	0.59	0.25	0.30	0.14	0.06	0.21	1				
Ti	0.24	0.28	0.23	0.12	0.19	0.37	0.30	0.54	−0.06	0.26	0.60	0.30	0.33	0.18	0.11	0.20	0.57	1			
V	0.20	0.09	0.12	0.21	0.31	0.08	0.21	0.15	0.08	0.06	0.16	0.08	0.15	0.11	0.09	0.31	0.12	0.20	1		
Zn	0.51	0.53	0.50	0.45	0.11	−0.01	0.37	0.20	0.15	0.23	0.37	0.45	0.23	0.03	0.31	0.15	0.14	0.20	0.17	1	
PM_2.5ext_^b^	0.85	0.52	0.60	0.64	0.30	0.01	0.52	0.21	0.18	0.24	0.36	0.48	0.20	0.08	0.27	0.34	0.13	0.22	0.23	0.52	1

a Includes six counties with species data, 2000–2003.

b Includes all nine counties in Table 1, 1999–2003.

**Table 4:** Summary of statistically significant associations between mortality and alternative pollutant lags (numbers in the table indicate whether single lags of 0–3 days were statistically significant).

	All-cause	Cardiovascular	Respiratory	Age > 65 years
PM_2.5c_^a^	—	1*, 3*	—	3**
EC	—	3**	—	—
OC	—	3*	—	—
NO_3_	0*	3*	—	0**
SO_4_	—	—	—	—
Al	—	—	—	—
Br	—	—	—	—
Ca	—	—	—	—
Cl	1*	—	—	1**
Cu	1**	—	3**	—
Fe	—	2*	—	—
K	—	2**	—	2*
Mn	—	—	—	—
Ni	—	—	—	—
Pb	—	—	—	—
S	—	—	—	—
Si	—	—	—	—
Ti	—	1*, 2*	3**	—
V	—	—	1*	—
Zn	—	3**	1*	1**, 3**
PM_2.5ext_^b^	0**, 1*	0**, 1**	1*, 2*	0**, 1**

The regression model includes time (4 df/year), 1-day lags of temperature and humidity (3 df), day of week, and pollutant.

a Includes six counties with species data, 2000–2003.

b Includes all nine counties in Table 1, 1999–2003.

* *p* < 0.10;

** *p* < 0.05.

**Table 5:** Summary of statistically significant associations between mortality and alternative pollutant lags during October–March, 2000–2003 (numbers in the table indicate whether single lags of 0–3 days were statistically significant).

	All-cause	Cardiovascular	Respiratory	Age > 65 years
PM_2.5c_^a^	3**	3**	—	3**
EC	3*	—	—	3*
OC	3*	3*	—	3*
NO_3_	—	3*	—	0*
SO_4_	0**, 3*	0*, 3**	—	0**
Al	—	—	—	—
Br	—	—	—	—
Ca	1**, 2*, 3*	—	—	3*
Cl	1**	1**	—	1**
Cu	1*	1**	—	—
Fe	2*, 3**	3**	—	3**
K	3**	—	—	3**
Mn	2**, 3*	3**	—	—
Ni	—	—	—	—
Pb	3**	—	—	3*
S	0*, 3**	3*	—	0**,3*
Si	1*, 3**	—	—	3**
Ti	1**, 2*, 3**	1**	—	3**
V	1**	3**	—	1**
Zn	3**	3**	1*	3**
PM_2.5ext_^b^	0**, 1**, 2**, 3**	0**, 1**, 2**, 3**	0**, 1**, 2**, 3**	0**, 1**, 2**, 3**

The regression model includes time (4 df/year), 1-day lags of temperature and humidity (3 df), day of week, and pollutant.

a Includes six counties with species data, 2000–2003.

b Includes all nine counties in Table 1, 1999–2003.

* *p* < 0.10;

** *p* < 0.05.
